# Malaria Data by District: An open-source web application for increasing access to malaria information

**DOI:** 10.12688/wellcomeopenres.15495.2

**Published:** 2019-12-02

**Authors:** Sean Tomlinson, Andy South, Joshua Longbottom

**Affiliations:** 1 Department of Vector Biology, Liverpool School of Tropical Medicine, Liverpool, L3 5QA, UK; 2 Centre for Health Informatics, Computing and Statistics, Lancaster University, Lancaster, LA1 4YW, UK

**Keywords:** Malaria, Open-access, Shiny, Application, R, Data accessibility

## Abstract

Preventable diseases still cause huge mortality in low- and middle-income countries. Research in spatial epidemiology and earth observation is helping academics to understand and prioritise how mortality could be reduced and generates spatial data that are used at a global and national level, to inform disease control policy. These data could also inform operational decision making at a more local level, for example to help officials target efforts at a local/regional level. To be usable for local decision-making, data needs to be presented in a way that is relevant to and understandable by local decision makers. We demonstrate an approach and prototype web application to make spatial outputs from disease modelling more useful for local decision making. Key to our approach is: (1) we focus on a handful of important data layers to maintain simplicity; (2) data are summarised at scales relevant to decision making (administrative units); (3) the application has the ability to rank and compare administrative units; (4) open-source code that can be modified and re-used by others, to target specific user-needs. Our prototype application allows visualisation of a handful of key layers from the Malaria Atlas Project. Data can be summarised by administrative unit for any malaria endemic African country, ranked and compared; e.g. to answer questions such as, ‘does the district with the highest malaria prevalence also have the lowest coverage of insecticide treated nets?’. The application is developed in R and the code is open-source. It would be relatively easy for others to change the source code to incorporate different data layers, administrative boundaries or other data visualisations. We suggest such open-source web application development can facilitate the use of data for public health decision making in low resource settings.

## Introduction

In recent years, the mapping of diseases has improved considerably in extent, resolution and accuracy ( Kraemer *et al.*, 2016). Increasingly, data and related spatial outputs are being made publicly available ( Briand *et al.*, 2018; Flueckiger *et al.*, 2015). However, the full potential of associated modelled outputs will only be realised if data are accessed and used to inform local decision making. Recent reviews have suggested that data repositories are mainly targeted toward researchers rather than decision makers and that there is a need to improve indicator data use in low- and middle-income countries ( Briand *et al.*, 2018; Omumbo *et al.*, 2013). We describe the development of an open-source web application, MaDD (Malaria Data by District) ( Tomlinson *et al*., 2019), that enables disease distribution data to be more accessible at a local level.

The Malaria Atlas Project (MAP) is an international consortium which provides geographical information on diverse aspects of malaria epidemiology ( Hay & Snow, 2006). The open-access data generated by MAP have the potential to influence policy at the national and subnational level ( Hay & Snow, 2006; Moyes *et al.*, 2013). The project includes sophisticated interpolation models that allow inference of malaria prevalence, as detailed in national and regional indicator surveys, at non-sampled locations ( Giorgi *et al.*, 2018; Hay & Snow, 2006). Getting contemporary estimates of malaria metrics to policy makers is essential, but barriers to acceptance exist, notably for modelled predictions; these include the complexity of the statistics described within output reports, and the description of assumptions made during the modelling process ( Whitty, 2015). Additional barriers include the sheer wealth of data available, making it difficult to find and choose data surfaces despite central repositories that may be easily navigable. These factors have contributed towards a general lack of modelled outputs being used by local-level implementation programmes in Africa ( Omumbo *et al.*, 2013).

Most modelled MAP data are provided as spatial estimates, presented as 5 × 5 km gridded surfaces, for example, estimates of *Plasmodium falciparum* prevalence and mortality, estimates of indoor residual spraying coverage and estimates of dominant vector species distributions and abundance ( Bhatt *et al.*, 2015; Gething *et al.*, 2016; Sinka *et al.*, 2016). Though data generated at this spatial resolution provides a visual indication of subnational disparities, it is not immediately clear how these data may be used directly in operational decision-making. For modelled data to be utilised by operational staff at a local level, there is a requirement for additional tools and the ability to convert such data into operationally useful metrics at the level of administrative units ( Knight *et al.*, 2016; Omumbo *et al.*, 2013; Whitty, 2015).

Data curated by MAP can already be accessed via online interactive maps ( Malaria Atlas Project, 2019), an online country profiles tool and the *malariaAtlas* R package ( Pfeffer *et al.*, 2018). These are powerful tools enabling access to MAP generated data that do include data summaries by administrative units. However, because of the wealth of data and functionality it is not straightforward to find and use these tools to perform district-level comparisons. Here, we present an application that allows rapid generation and comparison of summary statistics for a select suite of malaria indicator variables at the sub-national administrative level. MaDD is open-source and coded in R, so it can easily be modified to address local needs ( R Core Team, 2019). This is a step towards developing tools for local decision makers to inform questions such as, “where should we prioritise the targeting of IRS rounds this season?”.

## Methods

### Development background

Malaria and malaria-associated data from MAP are curated from a wide variety of sources, including national control programmes, national survey data, satellite imagery, published and grey literature ( Moyes *et al.*, 2013). Presently, MAP provide 93 data layers relating to malaria and associated metrics ( Pfeffer *et al.*, 2018). The data are mostly stored as gridded surfaces at a resolution of 1 or 5 km. To ensure that MaDD was easy to use and that users are not overwhelmed with the diverse range of MAP data, we refined the list of input data surfaces down to four impactful malariometric variables, these are:

1. Malaria incidence: all-age incidence rate (clinical cases per 1,000 population per annum) of *Plasmodium falciparum* malaria ( Bhatt *et al.*, 2015).
2. Malaria prevalence in children: age-standardised parasite rate for *Plasmodium falciparum* malaria for children two to ten years of age (PfPR2–10) ( Bhatt *et al.*, 2015).
3. Insecticide treated net coverage: proportion of the population who were protected by ITNs ( Bhatt *et al.*, 2015).
4. Travel time to nearest city: minutes ( Weiss *et al.*, 2018).

The data surfaces were chosen to illustrate the distribution of malaria incidence in the context of bed net distributions and proximity to cities. Other data layers could be added relatively easily by small modifications to the open source code. Surfaces were obtained from MAP utilising the ‘malariaAtlas’ R package (version 0.0.3) ( Pfeffer *et al.*, 2018). These surfaces were then aggregated using first-level administrative boundaries for each country in sub-Saharan Africa as provided by the Food and Agriculture Organization of the United Nations, and the ‘raster’ package (version 2.8-19) ( FAO, 2008; Hijmans, 2019). Boundaries for display were simplified to make the user interface quick and responsive to user choices (i.e. reduction of polygon features and aggregation of spatial resolution), using the ‘rmapshaper’ package (version 0.4.1) ( Teucher & Russell, 2018). Due to the computational time required to run zonal aggregation, this process was pre-computed, and the output data are stored with the application source code. We provide this preprocessing code within our open-source repository, so that other data layers from MAP or elsewhere can also be presented within the same framework.

### Implementation

We used R (version 3.5.0) and the Shiny (version 1.2.0) framework to develop an online user-interface which allows users to interact with African MAP data, providing summary statistics by subnational administrative units ( Chang *et al.*, 2018; R Core Team, 2019). Shiny allows interactive web applications to be created with R code. This enables the creation and modification of web applications by researchers without specific knowledge of application scripting languages and workflows, something that traditionally required a dedicated web developer. During development we made several choices regarding the user interface (UI) to ensure that the tool is user-friendly. The key goal that drove UI development was ease of use. The aim was to minimise the total number of inputs that the user was required to consider and maximise the space allocated for visualising the spatial surfaces and output report statistics. The UI for MaDD went through several iterations during development to reflect growing functionality and target audience feedback. Anticipated target audiences for MaDD are detailed in Table 1.

**Table 1.** Anticipated main interests for target audiences.

Target audience	Anticipated main interests
High-level policymakers	Prioritisation of the most burdensome administrative areas in which to roll out an intervention/control programme.
Donors	Maximising existing data accessibility to meet malaria eradication targets.
Technical health agencies (e.g. World Health Organization)	Monitoring of administrative-level trends across a suite of indicators, as a means of identifying operation efficacy.

### Operation

MaDD ( Tomlinson *et al*., 2019) can be publicly accessed at https://seantomlinson30.shinyapps.io/shiny-map-prize/, with any modern web browser (e.g. Firefox, Chrome, Safari or Edge). Though we will make efforts to ensure MaDD remains available on a public platform, this relies upon continued free server hosting being available. Interfacing with MaDD on a hosted platform allows computation and memory requirements to be handled by the hosting server, reducing the technical requirements for users. MaDD can also be run from the source code locally using R and RStudio. All source code for MaDD can be found under version control at https://github.com/SeanTomlinson30/SHINY-map-prize. The minimum system requirements of R and RStudio may change and can be found at https://www.r-project.org/ and https://www.rstudio.com/, respectively.

## Use case

**Figure 1.**
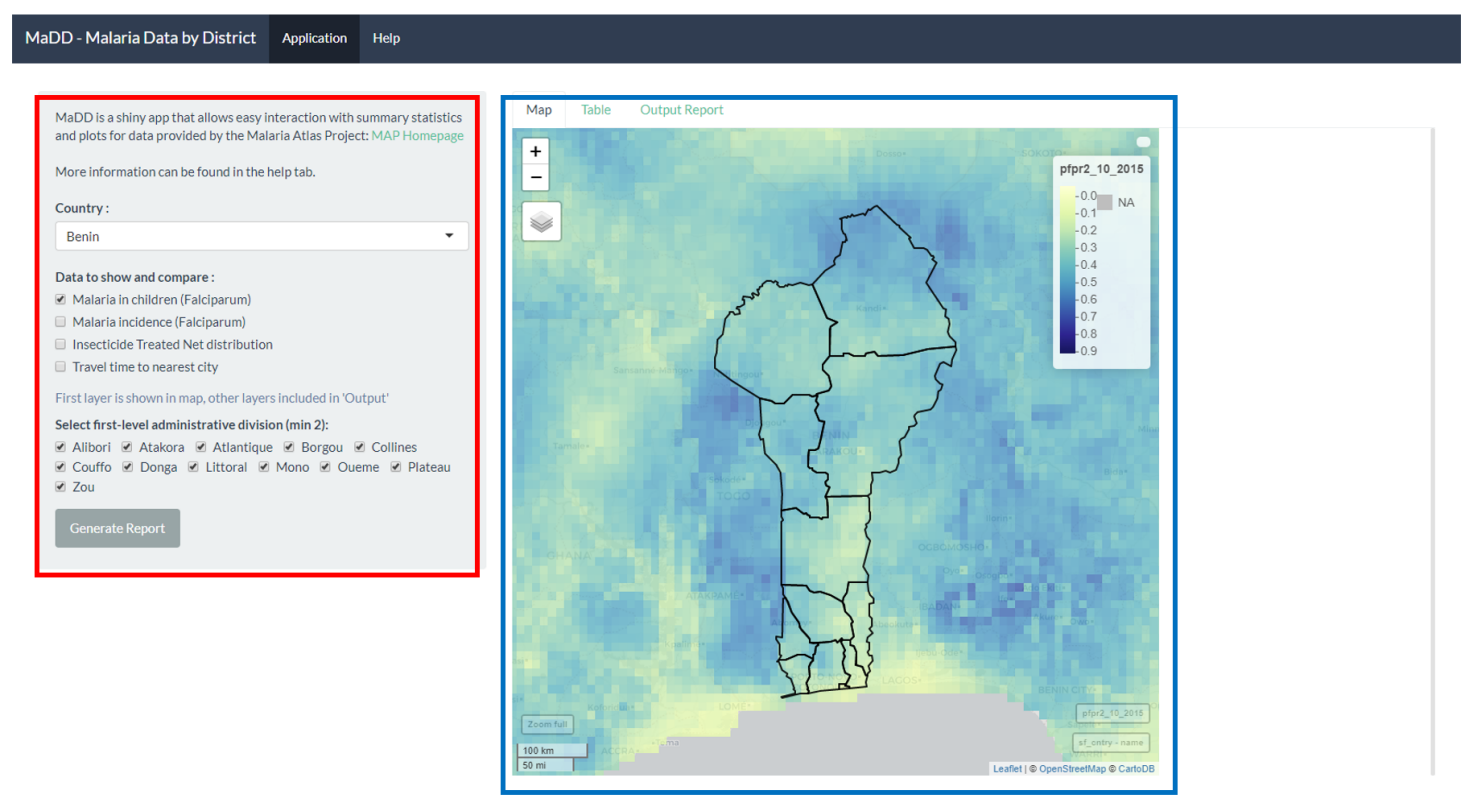
Screenshot of the Malaria Data by District application homepage. The main application page is split into a user input field (red box), which allows user to select the country, variable and districts of choice. The selected country is interactively plotted on the right side of the main page (blue box) and visualises the variables selected by the user.

Users can first select a country of interest. This selection is made from a list of malaria-endemic sub-Saharan African countries with modelled metrics, as provided in the scrollable “Country” field in the left-hand side panel of the MaDD UI ( Figure 1, red box). This country selection triggers an update of the map visible within the “Map” tab on the right-hand side of the application ( Figure 1, blue box). The “Map” tab is visible by default and dynamically updates to reflect the data surfaces the user has selected for comparison, as indicated by the “Data to show and compare” selection box within the input panel. Within this map, the user can hover the cursor over polygons (boundaries representing each administrative unit) to determine the name of the visualised administrative unit.

**Figure 2.**
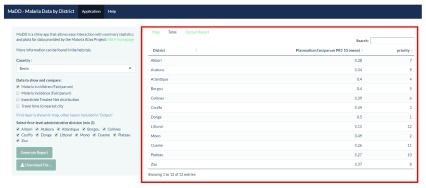
Screenshot of the Malaria Data by District application interactive table. Upon generation of the output file, an interactive table is populated (red box), which allows users to rank order data by column and search for variable names and statistics.

In the input section on the left, users can filter administrative divisions to compare; by default, all first-level administrative divisions for the country are selected. The mean values for all surfaces by administrative unit can be compared and ranked within the “Table” tab. This presents an interactive table ( Figure 2) that includes the priority (rank) for each variable and the ability to reorder the rows based on any column. Priority is set such that 1 indicates the “greatest need” i.e. highest malaria prevalence, lowest proportional coverage of bed nets and most remote district. This allows the user, for example, to order the rows based on malaria prevalence and then quickly see whether the administrative units with the greatest need also have other underperforming metrics; for example, a low proportional coverage of bed nets. There are likely to be other complicating factors influencing the interaction and causality across metrics, but this table allows users to determine where broad patterns are not as expected.

**Figure 3.**
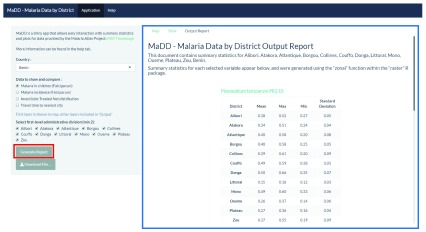
Screenshot of the Malaria Data by District application output report. Upon generation of the report (blue box), a download button becomes available (red box) allowing users to save the output file locally as an HTML file.

The “Generate Report” button present at the bottom of the left-hand side panel is used to generate summary statistics and country-specific choropleth maps, which are then shown in the “Output Report” tab ( Figure 3). These maps are also provided as a rendered RMarkdown document (saved locally as a temporary file on the user’s machine). Once the report is generated, a “Download Report” button becomes available, on the left-hand side panel, which enables the user to save the report as a locally viewable HTML file.

## Discussion

We present, MaDD ( Tomlinson *et al*., 2019) a prototype user interface demonstrating how MAP and other data can be made more accessible to local decision makers. MaDD permits easy access to MAP data at the level of administrative units, through administrative division aggregation, interactive tables and plots. MaDD has been developed with open-source software and can be easily edited to include additional data surfaces, calculations, visualisations, or expanded to focus on specific geographic regions.

One of the biggest challenges in developing successful digital tools is in getting them adopted and used ( Knight *et al.*, 2016). This has been recognised for digital projects in the development sector, prompting a set of Principles for Digital Development. These include recommendations that projects be designed with the user, understand the existing ecosystem, be collaborative and use open standards, open data, and open-source. Using open-source code (and particularly R given its wide adoption within a broad user community beyond computer scientists) has the advantage that tools can be adapted to fit local needs.

MaDD was developed to show summary statistics and plots for four impactful variables relating to malaria control; however, the framework we have developed is receptive to any of the surfaces generated by MAP. Though MaDD was developed for the African continent, future work could expand to include additional countries of interest, such as those with endemic malaria transmission in Asia or South America. Presenting publicly available data in an easily interactable and navigable way has the potential to increase public engagement and awareness of malaria trends to those concerned.

There are limitations to our application and approach. We aimed to demonstrate, with a prototype application, that open-source R software can be used to create useful and usable tools, improving access to data for malaria or other public health issues. However, we advise caution as this approach, and the application particularly, are not without risks. Firstly, focusing on the prototype application itself. We suggest that potential users critically assess whether the application and the data behind it, are appropriate to inform the questions they wish to address. For example, we would be comfortable with the application being used to give background on the malaria situation between administrative regions, but we would not be comfortable with operational decisions being made purely upon the rankings in one of the tables. One issue is the timeliness of the data layers. The process of data collection, modeling, layer creation and provision often takes years, such that publically available data may not be considered contemporary. Nevertheless, those data may be the best currently available and may still be useful to inform decision making in combination with other information sources.

On the issue of using other information sources, the prototype application deliberately had restricted options to promote usability. We recognise that the four data layers and single set of administrative boundaries we chose will not satisfy all potential users. There is a tension between usability and flexibility, i.e. making applications more flexible can make them more difficult to use and increases hosting requirements. Our aim was to show that highly usable applications can be created with open-source code. To move beyond the prototype, dialogue with users is required to establish what data layers and functionality are required and the open-source code can be modified accordingly. Such modification would not require a large software company or us but could be achieved by local R developers or data scientists. Since this work, we have won funding for a small project (afrimapr) to develop reusable R software building blocks, to make it easier to build applications like this with different data sources and functionalities. We will be working to promote the local development of open-source digital tools using R. These could, for example, use administrative boundaries available from national sources, which may be more recent or go to finer administrative levels. This will prompt other questions, such as the spatial resolution at which different global data layers can provide useful information.

We hoped that providing this demonstration and open-source code would allow others to create similar applications using data to inform local decision making for disease control. We were pleased to be recently contacted by the Data Integration team at USAID President's Malaria Initiative, who indicated they are integrating our application into their own malaria information platform (Okoko, personal communications). They are developing analytical tools to inform the geographic allocation of malaria resources by in-country staff and national malaria control programmes in 27 countries and have plans to adapt our code to their own needs. We look forward to this and other open tools being developed to improve the use of data for local decision making in public health.

## Data availability

The malariometric datasets analysed during this study are available from the Malaria Atlas Project database at: http://www.map.ox.ac.uk/explorer/, and the *malariaAtlas* R package ( Pfeffer *et al.*, 2018).

These data are available under the terms of a Creative Commons Attribution 3.0 license (CC BY 3.0).

## Software availability

MaDD application available at: https://seantomlinson30.shinyapps.io/shiny-map-prize/.

Source code available from: https://github.com/SeanTomlinson30/SHINY-map-prize.

Archived source code at time of publication: https://doi.org/10.5281/zenodo.3466884 ( Tomlinson *et al*., 2019).

License: MIT License.
