# Supplementary material for: Malaria Data by District: An open-source web application for increasing access to malaria information
Source: Wellcome Open Res. 2019 Dec 2;4:151. Originally published 2019 Oct 9. [Version 2] doi: 10.12688/wellcomeopenres.15495.2 (PMC6915811; doi:10.12688/wellcomeopenres.15495.2)
Supplement: Peer review history

# Peer review: Reviewer response for version 2

**DOI:** 10.21956/wellcomeopenres.17109.r37248

**Published:** 2019-12-16

**Authors:** Caroline A. Lynch

**Affiliations:** 1 Faculty of Epidemiology & Population Health, London School of Hygiene and Tropical Medicine, London, UK

I confirm that I have read this submission and believe that I have an appropriate level of expertise to confirm that it is of an acceptable scientific standard.

# Peer review: Reviewer response for version 2

**DOI:** 10.21956/wellcomeopenres.17109.r37247

**Published:** 2019-12-03

**Authors:** Peter Macharia

**Affiliations:** 1 Population Health Unit, Kenya Medical Research Institute - Wellcome Trust Research Programme, Nairobi, Kenya

I have no further comments.

I confirm that I have read this submission and believe that I have an appropriate level of expertise to confirm that it is of an acceptable scientific standard.

# Peer review: Reviewer response for version 1

**DOI:** 10.21956/wellcomeopenres.16953.r36714

**Published:** 2019-11-07

**Authors:** Caroline A. Lynch

**Affiliations:** 1 Faculty of Epidemiology & Population Health, London School of Hygiene and Tropical Medicine, London, UK

The authors describe an open-source, easily accessible application that has great potential for use by policy-makers or National Malaria Control Programmes deciding on how best to target interventions to ensure greatest coverage relative to burden of disease.

 My main comments are around the conclusions of the tool and its performance. The authors conclude that MaDD can present publicly available data in an easily interactable and navigable way. This is true. However, they also assert that this tool could inform local decision-makers asking questions such as ‘where should we prioritise the targeting of IRS rounds this season’. This highlights the fact that the limitations of the application are not fully discussed.

 Those limitations include timeliness of MAP revisions and incorporation of new prevalence data into models and ITN coverage data for example, relative to timing of decision-making at local levels. Is it true that a national policy maker could rely on this application to make year-on-year decisions as to where to target interventions and if not, then what would be required to allow that to happen?

 Another limitation related to above is the confidence with which estimates can be made at lower administrative levels. For example, how useful are the maps in DRC if they are only available at provincial level? If policy makers add in layers of lower administrative levels is there enough prevalence data to have the confidence to predict down to those levels?

 The authors correctly state that one of the biggest challenges in developing successful digital tools is getting them adopted and used. This tool is a streamlined simple tool which is really appealing, but the simplicity of it may mask the complexity of malaria epidemiology at sub-national levels and lead to a lack of confidence by users (e.g. National control programmes) of the data presented to some extent.

I confirm that I have read this submission and believe that I have an appropriate level of expertise to confirm that it is of an acceptable scientific standard, however I have significant reservations, as outlined above.

# Response

**Published:** 2019-11-29

**Authors:** Sean Tomlinson

**Affiliations:** Liverpool School of Tropical Medicine, UK

We thank Caroline Lynch for her insightful and practical comments that we have responded to below.

“The authors describe an open-source, easily accessible application that has great potential for use by policy-makers or National Malaria Control Programmes deciding on how best to target interventions to ensure greatest coverage relative to burden of disease.”

“My main comments are around the conclusions of the tool and its performance. The authors conclude that MaDD can present publicly available data in an easily interactable and navigable way. This is true. However, they also assert that this tool could inform local decision-makers asking questions such as ‘where should we prioritise the targeting of IRS rounds this season’. This highlights the fact that the limitations of the application are not fully discussed.”

**Response**: We agree that improved discussion of the limitations of the application and approach would be useful for the reader. We already assert that the application is a prototype and a step towards useful tools for local decision-makers, but we have added two paragraphs to the discussion expanding on the limitations of this prototype.

“Those limitations include timeliness of MAP revisions and incorporation of new prevalence data into models and ITN coverage data for example, relative to timing of decision-making at local levels. Is it true that a national policy maker could rely on this application to make year-on-year decisions as to where to target interventions and if not, then what would be required to allow that to happen?”

**Response**: This is an important point that limitations of decision-supporting applications and the data behind them need to be considered carefully. The timeliness of data is particularly relevant. We have presented a prototype and our aim is to show that useful decision-supporting applications can be built with open-source software. There is indeed a risk that applications could provide poor advice to decision-makers because of these issues. However, we suggest that such applications could make it easier to give decision-makers access to the currently-best-available data (than for-example commercial software applications or paper reports both of which could take longer than modifying an open-source application). We cover this in the new discussion paragraph 4.   

“Another limitation related to above is the confidence with which estimates can be made at lower administrative levels. For example, how useful are the maps in DRC if they are only available at provincial level? If policy makers add in layers of lower administrative levels is there enough prevalence data to have the confidence to predict down to those levels?”

**Response**: We also agree that care needs to be taken with the spatial resolution that data are aggregated to. In the future, we would like to see more guidance on the confidence that can be had in using such global, modeled datasets at a local level. We cover this in the new discussion paragraph 5.  

“The authors correctly state that one of the biggest challenges in developing successful digital tools is getting them adopted and used. This tool is a streamlined simple tool which is really appealing, but the simplicity of it may mask the complexity of malaria epidemiology at sub-national levels and lead to a lack of confidence by users (e.g. National control programmes) of the data presented to some extent.”

**Response**: We agree that there is a risk that local users may not trust applications such as this if they feel that it is over-simplified and does not represent the situation on the ground that they see. Our aim was to provide a prototype demonstrating that streamlined applications can be made. The beauty of open-source software is that if local users decide that we have gone too far and taken too much out, it is relatively straightforward to add additional data or functionality. Such modification would not require paying a northern hemisphere software company (or us) more money, but instead could be done by local data-scientists or R developers. We cover this in the new discussion paragraph 5.

# Peer review: Reviewer response for version 1

**DOI:** 10.21956/wellcomeopenres.16953.r36713

**Published:** 2019-10-10

**Authors:** Peter Macharia

**Affiliations:** 1 Population Health Unit, Kenya Medical Research Institute - Wellcome Trust Research Programme, Nairobi, Kenya

The authors describe an application that has a big potential, especially for policy makers to make informed decisions by making public health-related datasets/maps easily accessible via an online platform. My main comments include allowing for more flexibility for the end-user (allow the user to pre-load a shapefile of choice or present more options) and incorporation of more public health-related datasets.

1. While refining MAP data to four products makes the app easily navigable, what of other users who might be interested in other products and not necessarily the four that were chosen for MaDD? 
2. Are there plans to extend the app and have more products? There are more spatial products in the public domain that would benefit from being repackaged at a subnational scale for district managers. For example, the population distribution maps, gridded surface showing travel time to different tiers of healthcare. This is because the end users of the app are mainly policy makers as opposed to researchers. 
3. The subnational boundaries are dynamic and vary by size, shape and number within a country. It would be important for the policy makers to be able to load different sub-national units within the app or add functionality to the app to load boundaries from other sources ( GADM, HDX, DIVA-GIS and others).
4.  It is also often the case that a country has different administrative boundaries at level 2, 3 and 4 and users would be interested at lower scale variation if they are district managers. For example, under the current implementation of MaDD, Kenya is shown by its 8 former provinces while the current structure is 47 sub national units (counties) representing 47 county governments which are further broken down to sub counties and wards.

I confirm that I have read this submission and believe that I have an appropriate level of expertise to confirm that it is of an acceptable scientific standard, however I have significant reservations, as outlined above.

# Response

**Published:** 2019-11-29

**Authors:** Sean Tomlinson

**Affiliations:** Liverpool School of Tropical Medicine, UK

We thank Peter Macharia for his detailed and helpful comments that we have responded to below.

Response to 1: This is a good point. We recognise that the four data layers we chose will not satisfy all potential users. There is a tension between usability and flexibility. Our aim was to show that highly usable applications can be created with open-source code. Our application is a prototype. Dialog with users is required to establish where additional data layers are required and then the open-source code can be modified to incorporate them. We include this text in two paragraphs we have added to the discussion.

Response to 2: In short, yes, there are plans to extend this work. Specifically, we have won funding to develop reusable R software building blocks to make it easier to build applications like this with different data sources and functionalities. We added mention of this to the discussion paragraph 5.

Response to 3: We agree that applications should be able to use boundaries from different sources. In this prototype, we used a single set of boundaries to maintain high usability. As we provide the source code it would be relatively easy for developers to use different boundaries. A primary aim of our upcoming project, to develop reusable R building blocks for spatial data in Africa, is to make it easier to use different boundary data sets. 

Response to 4: An excellent point that sub-national boundaries are constantly changing and that it is difficult for global public data providers to keep up-to-date. Countries often have finer scale sub-national and administrative boundaries. When designing applications for national-level decision making it should be possible to use these other national data sets. Flexible application design could allow default pre-loaded boundaries to be used with the option to load other boundaries if they are available. We added mention of this to the discussion paragraph 5.
